# Fronto-central slow cortical activity is attenuated during phasic events in rapid eye movement sleep at full-term birth

**DOI:** 10.1016/j.earlhumdev.2019.07.007

**Published:** 2019-09

**Authors:** Kimberley Whitehead, Maria Slobodina, Judith Meek, Lorenzo Fabrizi

**Affiliations:** a Department of Neuroscience, Physiology and Pharmacology, University College London, London WC1E 6BT, United Kingdom; b Elizabeth Garrett Anderson Wing, University College London Hospitals, London WC1E 6BD, United Kingdom

**Keywords:** Neonatal, Active sleep, EEG, Oscillations, Tonic, Wakefulness

## Abstract

Delta and theta power across fronto-central regions is lower during phasic (saccadic eye movements) than tonic rapid eye movement (active) sleep in full-term infants (*n* = 15). This indicates that the behavioural-electrophysiological pillars of rapid eye movement sleep micro-architecture are in place at birth.

## Highlights

• Slow cortical activity is lower during phasic than tonic REM sleep in neonates.
• This attenuation is localised to the fronto-central regions.
• REM sleep micro-architecture is in place at full-term birth.
• Results provide insight into the spatial dynamics of the newborn brain.

## 1 Introduction

Rapid eye movement (REM) sleep is a unique behavioural state, especially for its hallmark saccades. However, these movements occur intermittently (for approximately 20% of the time), and therefore REM sleep can be divided into two sub-states: phasic REM sleep in which saccades occur (in phases), and tonic REM sleep in which saccades are not present but all other features of REM sleep - such as irregular breathing rate - are. During phasic REM sleep, sensory thresholds to the environment are higher [1], ability to encode sensory information is lower [2], and cortical oscillations have lower theta and alpha-beta power, compared to tonic REM sleep [[3], [4], [5]].

Newborns spend up to half of the day in REM sleep (sometimes termed active sleep in this population) and also exhibit intermittent saccades during this state [6]. However, it is not known whether phasic REM sleep is accompanied by modulation of cortical activity relative to tonic REM sleep, as in adults. As brain activity is already related to the *overall* sleep-wake cycle in full-term infants [7,8], we hypothesised that it would also differ between the phasic and tonic sub-states of REM sleep. To address this, we compared the power content of electroencephalography (EEG) recordings between phasic and tonic REM sleep. To test whether differences were specific to phasic REM sleep, rather than general to all states featuring saccades including wakefulness, we also performed the same comparison between wakefulness and tonic REM sleep.

## 2 Material and methods

### 2.1 Subjects

Fifteen full-term infants (median 41 + 1 weeks + days corrected gestational age, range 39 + 0–42 + 6; six female) with median postnatal age five days (range 1–11) were included. Corrected gestational age is defined as gestational age at birth plus postnatal age. Infants were recruited from the postnatal and special care wards at the Elizabeth Garrett Anderson wing of University College London Hospitals. No infants required EEG for clinical purposes. No neonates were acutely unwell, receiving neuroactive medication (including caffeine), or respiratory support at the time of study. All neonates were neurologically normal both at the time of study and at the date of discharge, and were considered at low risk of adverse neurodevelopment, based on review of medical notes and the discharge summary. All EEGs were assessed as normal for corrected gestational age by a clinical scientist (KW) according to standard criteria, and presence of appropriate sleep architecture including REM-onset sleep [[9], [10], [11]]. Ethical approval was obtained from the NHS Research Ethics Committee, and informed written parental consent was obtained prior to each study. Additional written parental consent was obtained to publish video data from one infant.

### 2.2 Recordings

**Fig. 1:**
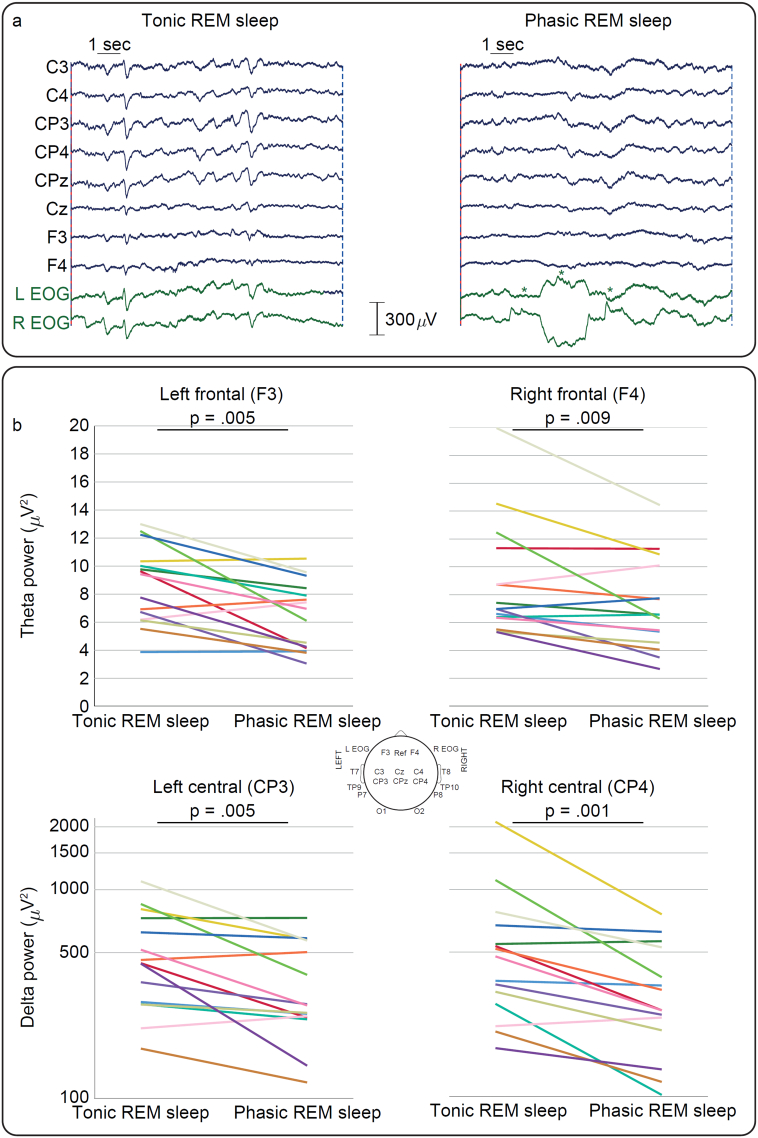
Cortical activity during tonic and phasic REM sleep. a: Examples of tonic and phasic REM sleep epochs in the same infant. EOG = electrooculography. Each horizontal saccade is marked by an asterisk. Only fronto-central EEG channels are displayed for clarity. b: Theta power (linear-scaled) and delta power (log-scaled) are lower in phasic than tonic REM sleep. Each coloured line represents one infant.

Sixteen EEG electrodes were positioned bilaterally overlying frontal (F3, F4), central (C3, C4, CP3, CP4, CPz, Cz), temporal (T7, T8, TP9, TP10, P7, P8) and occipital scalp areas (O1, O2). On 2/15 occasions a reduced number of electrodes (two and four less) were used because the infant became slightly unsettled. The reference electrode was placed at Fz. Electrooculography (EOG) was recorded from electrodes placed laterally to the eyes: these capture corneo-retinal dipole potentials, as well as some EEG activity (Fig. 1a). Lead I electrocardiography (ECG) was recorded from both shoulders. Respiratory movement was monitored with a transducer at the thorax. EEG was recorded with a direct current (DC)-coupled amplifier from DC-800 Hz using the Neuroscan (Scan 4.3) SynAmps2 EEG/EP recording system. Signals were digitized with a sampling rate of 2 kHz and a resolution of 24 bit. The median EEG recording length was 49 min (range 32 to 70), and recordings commenced between 10:00 and 18:00.

### 2.3 Data analysis

Data analysis was carried out using EEGLAB v.14 (Swartz Center for Computational Neuroscience), custom-written Matlab code and IBM SPSS version 25. Mains interference was removed with a 50 Hz notch filter (4th order Butterworth filter). Missing recordings were estimated with spherical interpolation as implemented in EEGLAB. Periods of REM sleep were first identified at the cot side - closed eyes with intermittent saccades and largely irregular breathing - and confirmed offline by assessing the intermittent presence of saccades (EOG), largely irregular respiratory rate and depth (transducer at thorax), and overall relatively low amplitude continuous EEG (compared to non-REM sleep [8] (sometimes termed quiet sleep in neonates)), according to the criteria of the American Academy of Sleep Medicine [10]. Wakefulness was defined by cot side observation of continuously wide-open eyes [10]. Twelve-second artefact-free REM sleep and wakefulness epochs, during which the infants were not undergoing any stimulation, were extracted for analysis. This is an appropriate epoch length because it contains over two cycles of our lowest frequency of interest (0.2 Hz) [12]. Each REM sleep epoch was subcategorised: phasic epochs were defined by the presence of at least three horizontal saccades; tonic epochs did not contain any saccades (Fig. 1a). The median number (range) of epochs included for each infant was: tonic REM sleep = 8 (1–28), phasic REM sleep = 2 (1–5), wakefulness = 5 (2–7). For each epoch, DC offset was removed and the power spectrum (μV^2^) was calculated for each channel, using a Hanning window to reduce spectral leakage. This was then averaged across all the epochs within a state for each infant, leading to a single power spectrum per state per subject. We compared the power content in the delta (0.2–4 Hz), theta (4–8 Hz), and alpha-beta (8–20 Hz) frequency bands between tonic and phasic REM sleep. We then compared the power in the same frequency bands between tonic REM sleep and wakefulness in a subset of 5/15 infants in whom this latter state occurred during the recording. Statistical significance threshold was set to 0.05 for all tests. Data were analysed with non-parametric tests because they were not normally distributed (Shapiro-Wilk test <.05). Wilcoxon paired tests were used to compare EEG power content between sleep-wake states within-subject.

## 3 Results

Delta power was lower during phasic than tonic REM sleep for the central regions predominantly (C4 *p* = .001; CP4 p = .001; C3 *p* = .036; CP3 *p* = .005; Cz p = .001; O1 *p* = .027 (Fig. 1b); all other electrodes *p* ≥ .061]. Theta power was lower during phasic than tonic REM sleep for the fronto-central regions (F4 *p* = .009; F3 p = .005; C3 *p* = .012; Cz *p* = .031 (Fig. 1b); all other electrodes ≥ .078). These regional changes were specific to these slow frequencies (no changes in alpha-beta power (*p* ≥ .069)).

During wakefulness - a state which also features saccades (Supplementary Fig. 1) - delta power was *higher* than tonic REM sleep across parts of the central region (C4 and Cz both *p* = .043), and theta power was not significantly different (*p* ≥ .138).

## 4 Discussion

We demonstrate here that resting brain rhythms during REM sleep are organised by behavioural micro-structure at full-term birth. Behavioural sleep cycling emerges early in gestation: periods of quiescence (non-REM sleep) are interspersed with active periods characterised by intermittent saccades (REM sleep) from 23 to 27 weeks in foetuses [[13], [14], [15]] and extremely pre-term infants (Video 1) [16]. Two of the most prominent early developmental changes in sleep-wake behaviour are increasing wakefulness, which by full-term age is cyclically organised around feeds on demand [7,11], and then the emergence of skeletal muscle atonia during REM sleep throughout the course of the first year [10,17,18].

Sleep-wake behaviours begin to align with cortical activity patterns in pre-term infants from 31 to 34 weeks, when REM sleep and wakefulness can be clearly dissociated from non-REM sleep by relative continuity of EEG activity [7,11,19,20]. Subsequently, from full-term age REM sleep can be distinguished from wakefulness by its slightly lower delta power [8,21,22]. Finally, at 2 months of age adult-like state-specific electrophysiological features emerge, such as sleep spindles during non-REM sleep [10]. (As the complete spectrum of behavioural and electrophysiological sleep-wake indices are establishing during infancy, non-REM and REM sleep are sometimes described as quiet and active sleep respectively in neonates).

Here we show that at full-term birth neural activity is already organised in sub-states within REM sleep, by demonstrating that delta and theta power is attenuated during phasic compared to tonic periods of the sleep state. This attenuation of cortical activity cannot be an artefact caused by the shift of corneo-retinal dipole potentials, as saccades are associated with a *decrease* in recorded activity rather than an increase; neither is it simply associated with eye movements per se: in wakefulness – within which saccades are also present – slow cortical rhythms over the central regions are *enhanced* relative to tonic REM sleep. Similarly, pyramidal neurons decrease their firing rates during phasic REM sleep in cats, but saccades during wakefulness are not associated with the same effect [23]. Saccades and the overall low amplitude of the EEG in REM sleep are mediated by distinct neuronal populations within the reticular formation of the brainstem [24]. Although discrete, these neuronal populations are functionally interconnected [25], perhaps explaining co-variation of eye movements and electrographic signs.

In adult humans and animals, attenuation of neural activity during phasic REM sleep is localised to primary sensorimotor cortex, and resembles the attenuation associated with voluntary movement in wakefulness [3,26,27]. In humans it has therefore been suggested to be related to internal generation of the active dream content recalled by adult subjects when awakened from phasic REM sleep [3,28]. The fronto-central distribution of the attenuation observed here (i.e. over sensorimotor cortex) could then relate to a similar phenomenon. In early life, patterning of somatomotor cortical activity synchronises functional ensembles, which subserves the refinement of somatosensation and resultant control of voluntary movement [[29], [30], [31], [32]]. Therefore understanding factors which regulate this patterning – including sleep microstructure – sheds light on this crucial aspect of brain development [8,[33], [34], [35]].

This study has some limitations. Given that the sample size is relatively small, we have studied a tight age group of full-term infants. Therefore, our results cannot necessarily be extrapolated to other stages of development. Future EEG studies should be conducted across a full 24-hour period in a larger population of full- and pre-term infants, in order to shed further light on the ontogeny of sleep-wake architecture – one of the earliest organising principles of the complex neural activity patterns of the newborn.

The following are the supplementary data related to this article.

Video 1 Example of saccades during rapid eye movement sleep in an infant with corrected gestational age 30 + 6 weeks + days. Video 1

Supplementary Fig. 1 Cortical activity during saccades in wakefulness. Example of a wakefulness epoch. EOG = electrooculography. Examples of horizontal saccades are marked by an asterisk. Only fronto-central EEG channels are displayed for clarity. Supplementary Fig. 1

## Author contributions

KW: conception, data collection, analysis and manuscript preparation. MS: preliminary analysis and manuscript review. JM: supervision of data collection and manuscript revision. LF: analysis and manuscript revision.

## Declaration of Competing Interest

None declared.
