# Supplementary material for: Fronto-central slow cortical activity is attenuated during phasic events in rapid eye movement sleep at full-term birth
Source: Early Hum Dev. 2019 Sep;136:45–8. doi: 10.1016/j.earlhumdev.2019.07.007 (PMC6697120; doi:10.1016/j.earlhumdev.2019.07.007)
Supplement: Supplementary Fig. 1 — Cortical activity during saccades in wakefulness. Example of a wakefulness epoch. EOG = electrooculography. Examples of horizontal saccades are marked by an asterisk. Only fronto-central EEG channels are displayed for clarity. [file mmc2.docx]

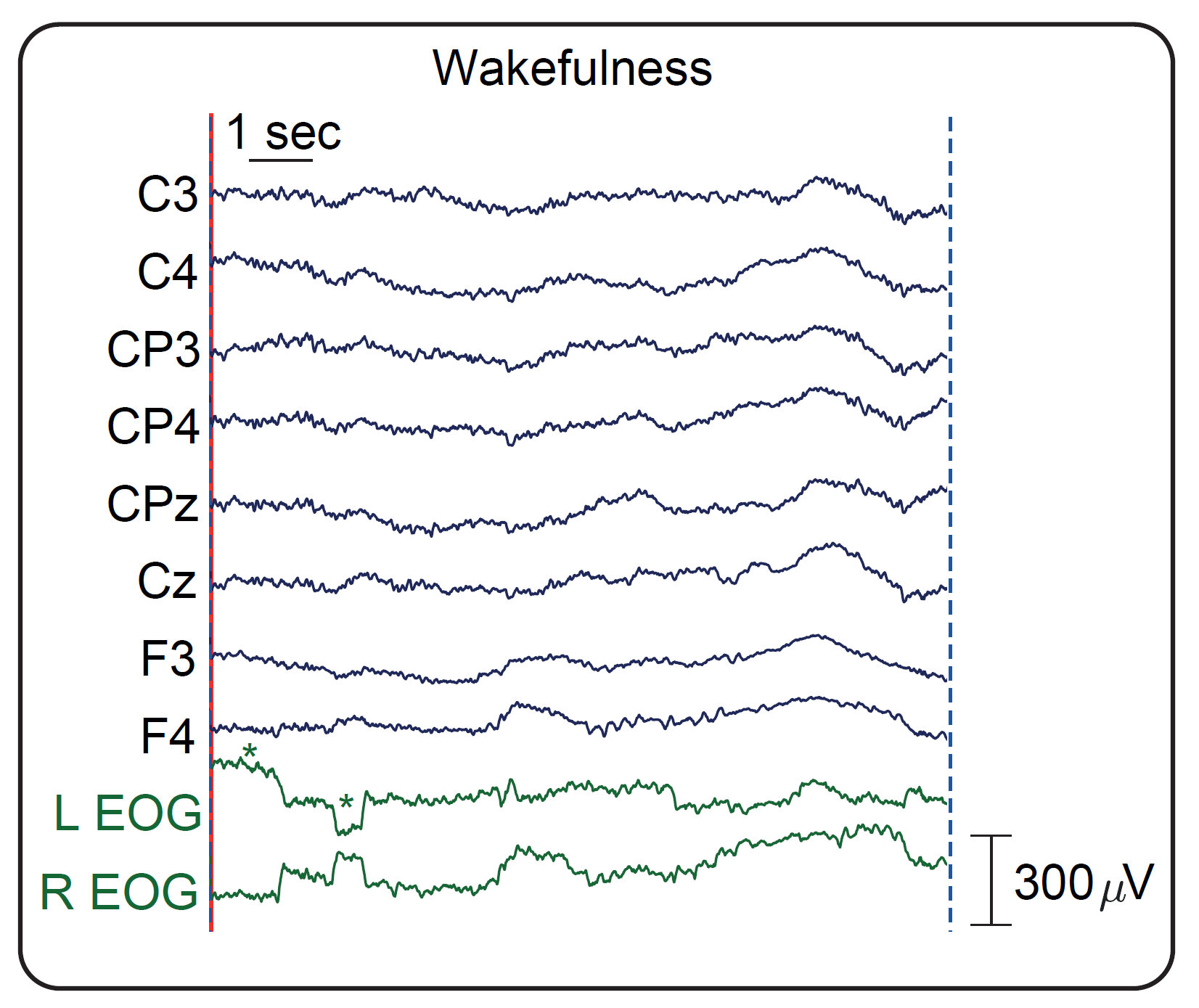


**Supplementary Figure 1: Cortical activity during saccades in wakefulness.** Example of a wakefulness epoch. EOG = electrooculography. Examples of horizontal saccades are marked by an asterisk. Only fronto-central EEG channels are displayed for clarity.
